# Micronutrient Deficits Are Still Public Health Issues among Women and Young Children in Vietnam

**DOI:** 10.1371/journal.pone.0034906

**Published:** 2012-04-17

**Authors:** Arnaud Laillou, Thuy Van Pham, Nga Thuy Tran, Hop Thi Le, Frank Wieringa, Fabian Rohner, Sonia Fortin, Mai Bach Le, Do Thanh Tran, Regina Moench-Pfanner, Jacques Berger

**Affiliations:** 1 Global Alliance for Improved Nutrition (GAIN), Geneva, Switzerland; 2 National Institute of Nutrition (NIN), Hanoi, Vietnam; 3 Institut de Recherche pour le Développement (IRD), UMR Nutripass IRD-UM2-UM1, Montpellier, France; Oklahoma State University, United States of America

## Abstract

**Background:**

The 2000 Vietnamese National Nutrition Survey showed that the population's dietary intake had improved since 1987. However, inequalities were found in food consumption between socioeconomic groups. As no national data exist on the prevalence of micronutrient deficiencies, a survey was conducted in 2010 to assess the micronutrient status of randomly selected 1526 women of reproductive age and 586 children aged 6–75 mo.

**Principal Findings:**

In women, according to international thresholds, prevalence of zinc deficiency (ZnD, 67.2±2.6%) and vitamin B12 deficiency (11.7±1.7%) represented public health problems, whereas prevalence of anemia (11.6±1.0%) and iron deficiency (ID, 13.7±1.1%) were considered low, and folate (<3%) and vitamin A (VAD, <2%) deficiencies were considered negligible. However, many women had marginal folate (25.1%) and vitamin A status (13.6%). Moreover, overweight (BMI≥23 kg/m^2^ for Asian population) or underweight occurred in 20% of women respectively highlighting the double burden of malnutrition. In children, a similar pattern was observed for ZnD (51.9±3.5%), anemia (9.1±1.4%) and ID (12.9±1.5%) whereas prevalence of marginal vitamin A status was also high (47.3±2.2%). There was a significant effect of age on anemia and ID prevalence, with the youngest age group (6–17 mo) having the highest risk for anemia, ID, ZnD and marginal vitamin A status as compared to other groups. Moreover, the poorest groups of population had a higher risk for zinc, anemia and ID.

**Conclusion:**

The prevalence of anemia and ID in Vietnam has been markedly reduced over the last decade, but a large part of the population is still at risk for other deficiencies such as zinc, vitamin A, folate and vitamin B_12_ especially the youngest children aged 6–17 mo. Consequently specific interventions to improve food diversity and quality should be implemented, among them food fortification of staple foods and condiments and improvement of complementary feeding.

## Introduction

A recent review of dietary intake data from Asia showed that recommended daily intakes of many essential micronutrients, such as vitamin A, iron, calcium, riboflavin, zinc and iodine, were not met [1]. This is also the situation for Vietnam where besides micronutrient intake, macronutrient intake is also too low [2]. Indeed, the food consumption survey conducted in 2009 showed that 20% of the Vietnamese population was not meeting its energy requirements (personal communication), against 40% in 2005 [2]. In 2005, the energy intake comes from a predominantly rice based diet, with an average rice consumption of over 350 g/d [2]. This diet also results in low intakes of micronutrients with over 70% of Vietnamese population consuming less than the recommended amounts of most micronutrients [2]. Over the last decade, Vietnam experienced a period of rapid economic growth [3] and a reduction in poverty from 37.4% in 1998 to 16% in 2006 [4], and to 13.4% in 2008 [5]. In the same time, nutrient intakes, especially vitamins, increased significantly [2].

In parallel to dietary intake data, nutritional status as measured by anthropometry has improved over the last decades but remains of concern. The prevalence of underweight among children under 5 years of age declined from 45% in 1990 to 26.6% in 2004 [6] and the prevalence of stunting decreased from 60% in 1985 to 30% in 2005 [7]. Moreover, recent intervention pilot studies showed that the prevalence of anemia ranges from 25% to 45% in schoolchildren [8], [9] and 24% in women of reproductive age [10]. In addition, the prevalence of zinc and selenium deficiencies were also reported to be very high (>50%) in rural Vietnamese children [11]. However, another study carried out in 2008 in two Vietnamese provinces of the Mekong of Delta among women of reproductive age did not show evidence of folate and vitamin B_12_ deficiencies, although many women had marginal folate status [11].

Over the last 10 years, a number of trials conducted in Vietnam have demonstrated the efficacy of micronutrient fortification of fish sauce [10], [12], milk [9], biscuits [8], [13], complementary food [14], instant noodles [15], [16] and of micronutrient supplements [17], [18], [19], to reduce micronutrient deficiencies among several population groups (infants, preschool and school-aged children, and women of reproductive age).

Controlling micronutrient deficiencies has been one of the priorities for the Vietnamese government in the 2001–2010 National Nutrition Strategy and is still a priority in the 2011–2020 National Nutrition Strategy. However, to develop a comprehensive national plan of interventions, there is an urgent need for national data on vitamin and mineral deficiencies. In 2009 a nationwide food consumption survey (FCS) was carried out among young children and women of reproductive age. The present study was carried-out on a subset of households from the FCS that were re-surveyed in 2010 to provide additional information on iron, zinc, vitamin A, vitamin B12 and folate status of women in reproductive age and young children, in relation to their living area (urban/rural) and socio-economical status.

## Materials and Methods

### Study design and sampling

The survey population of the 2009 FCS consisted of 7680 households (HH), sampled from 512 clusters (104 urban and 408 rural). HH were randomly selected using a stratified 2-stage cluster sampling procedure with probability proportionate to size (PPS). A subset of HH from the FCS was selected for this 2010 micronutrient study (MNS). The sample size for this study was estimated on the basis of a prevalence of anemia among women of reproductive age of 50% as in the latest anemia national survey implemented in 1995, 39.9% of the non-pregnant and 52.5% of pregnant women were anemic [20]. Therefore, a sample size of 694 women per stratum (urban/rural) was calculated to get a precision of 5.0% with an expected design effect of 2.0. Anticipating an estimated 17% refusal or absence of women in the selected HH, 840 HH per stratum were required. Consequently a total of 56 urban and 56 rural clusters of 15 households were selected for this study. Every woman and every child from 6 months to 7 years of the selected households were surveyed. With an estimation of 0.28 child per household in the age range of 6–59 months, the study planned to recruit at least 470 children. Due to limited funding, 19 provinces were first selected by NIN at random on the list of the 64 provinces to pick 56 urban clusters from those selected for the 2009 FCS. Thereafter, 56 rural clusters also from those selected for the 2009 FCS were randomly selected in the same 19 provinces. Selected households were incorporate in the study with assistance of local guides who were involved in the 2009 FCS.

### Inclusion and exclusion criteria

The women of reproductive age were selected according to the following criteria: i) Living in households having participated in the 2009 food consumption survey; ii) Non-pregnant women aged 15–49 years; iii) No reported severe or chronic illness; iv) No reported or diagnosed current fever, diarrhea, ARI, or other acute infections; v) Having signed the informed consent form to participate.

The criteria for children less than six years to participate in the survey were as follows: i) From households having participated in the 2009 food consumption survey; ii) No reported severe or chronic illness, including congenital abnormalities, mental or severe physical handicap; iii) No reported or diagnosed current fever, diarrhea, ARI, or other acute infections; iv) Written informed consent signed by at least one parent.

### Anthropometry

Selected women and children were invited to come to the Commune Health Center early morning. Children and women were weighed without shoes or sandals and wearing light clothes by using a balance with a precision of 0.1 kg (Body Composition Monitor Scale Tanita BC-543, Japan). Height was measured by using a height measuring device with a precision of 0.1 cm (wooden height board, UNICEF). For children under two years, measurement of recumbent length was taken on an adjustable child length measuring board with a precision of 0.1 cm (wooden length board, UNICEF).

Anthropometric data were entered through EpiData (version 6.0, CDC). For children from 6 months to 6 years, anthropometric z-scores were calculated using the National Center for Health Statistics/WHO growth reference data of 2006 [21], using the Access Visual Basic program incorporating tables from the WHO Anthro software for standard population. Anthropometric status was assessed by the following indicators: weight-for-age<-2z-scores (WAZ) for underweight, height-for-age<-2 z-scores (HAZ) for stunting and weight-for-height<-2 z-scores (WHZ) for wasting.

For women, Body Mass Index (BMI) was calculated as the body weight (in kg) divided by the square of height (in meters). Women were classified using BMI cut-off points endorsed by WHO as underweight (BMI<18.5 kg/m^2^), normal (BMI of 18.5–24.9 kg/m^2^), overweight (BMI≥25 kg/m^2^) and obese (BMI ≥30 kg/m^2^) [22]. As other specific cut-offs have been suggested for Asian populations a BMI of 23–27.49 kg/m^2^ was also used for overweight and a BMI ≥27.5 kg/m^2^ for obesity [23].

### Blood sampling and analysis

Just after anthropometry, a non-fasting blood sample was collected between 0700 and 0900 hours from women and children from the selected HH. Venous blood, 6 ml for women and 4 ml for children, was drawn by venipuncture in trace-element free heparinized sampling tubes (Vacuette, Greiner Bio One, Austria) by licensed medical technicians at the health center of the commune. Blood samples were stored in the dark in a cool box and transported within 4 hours of collection to the laboratory of Provincial Medical Center (PMC) for hemoglobin determination. Hemoglobin concentration (Hb) was then measured immediately using the HemoCue device (Hemocue 301, Ängelholm, Sweden) and control material provided by Hemocue and according to the manufacturer's instructions. Thereafter, plasma was obtained by centrifugation at 3000 g for 10 min at 4°C. Plasma was aliquoted into 200 µL pre-labeled Eppendorf tubes and kept frozen at −20°C at the PMC before being sent (within two weeks) on dry ice to the National Institute of Nutrition (NIN) where samples were stored at −70°C until analysis.

Plasma concentrations of ferritin (PF), retinol (PR), zinc (ZN), and C-reactive protein (CRP) were determined at the Micronutrient Laboratory of NIN. PF was measured by ELISA using commercial kits that included reference samples (Ramco Laboratories Inc, Houston, Texas). The accuracy was tested with Bio-Rad liquicheck control (Bio-Rad Laboratories, USA). PR concentration was determined by reverse-phase HPLC (LC-10 ADVP, Shimadzu, Japan) according to the method of the International Vitamin A Consultative Group in a dimly lighted room [24]. ZN was analyzed using a flame atomic absorption spectrophotometer (GBC, Avanta+) using trace element-free procedures, and powder free gloves (Latex Surgical Glove), and results were verified using reference materials (Liquicheck, Bio-Rad Laboratories, USA). A control for potential contamination of material used for blood sampling and blood treatment by external zinc was carried out by analyzing zinc content of at least 20 sets of material (needle, syringe, vacutainers and eppendorf tubes) where blood was replaced by bi-distilled water. CRP, an acute phase protein was used to quantify the acute phase response as a marker of inflammation and infection. Plasma CRP was measured by ELISA using a commercial kit from GenWay (Biotech, Inc., San Diego, California), with reference materials included in each assay (Liquicheck, Bio-Rad Laboratories, USA).

Aliquots of plasma were sent to the Swiss Vitamin Institute (Epalinges, Switzerland) for analysis of folate and vitamin B_12_ using a microbiological assay. For vitamin B_12_, a random plasma sample of 505 women of reproductive age was selected. For pre-school children, a random plasma sub-sample of 268 for folate analysis was selected but vitamin B_12_ was not analyzed. Extracted folates were diluted with basal medium containing all required growth nutrients except folate [25]. The growth response of *Lactobacillus casei*, subspecies rhamnosus (ATCC 7469) to extracted folates was compared to the growth response to calibration solutions with known concentrations. Vitamin B_12_ was analyzed turbidimetrically using the growth of *Lactobacillus plantarum* (ATCC 8014) as test organism [26], [27], [28]. The growth response of the organism is proportional to the quantity of vitamin B_12_ [29]. The within-assay variability for plasma ferritin, retinol, folate, zinc, vitamin B_12_, and CRP was <6%.

Anemia was defined according to WHO standards as Hb below 110 g/l for children aged from 6 months to 5 years, below 115 g/l for children from 5 to 11 years and 120 g/l for women of reproductive age [30]. Iron deficiency (ID) was defined as PF <12 µg/l for children 6–59 months and PF <15 µg/L for children above 5 years of age and women [30] and using a correction factor of 0.65 for ferritin concentration from subjects with sub-clinical inflammation as indicated by increased concentrations of CRP (>5 mg/l) [31]. Iron deficiency anemia (IDA) was defined as the combination of anemia and ID [30]. Sub-clinical vitamin A deficiency was defined using WHO cut-offs as PR <0.7 µmol/l [30] while marginal status was defined as population with a PR between 0.7 and 1.05 µmol/l [32] and plasma retinol concentrations were corrected where sub-clinical infection existed [33]. Zinc deficiency was defined using the International Zinc Nutrition Consultative Group (IZiNCG) cutoffs by ZN <9.9 µmol/l for children and ZN<10.1 µmol/l for women [34]. Vitamin B_12_ deficiency was defined as plasma vitamin B_12_ values below 148 pmol/l, and marginal status when the concentration was between 148–220 pmol/l [35]. Folate deficiency and marginal status were considered when plasma folate was respectively below 6.8 nmol/l and between 6.8–13.4 nmol/l [35].

### Socioeconomic status

Socio-economic status was calculated from data obtained during the 2009 FCS [36], using the Demographic Health Statistic (DHS) Wealth Index to divide households surveyed into five socio-economic quintiles: the “extreme poor” (category 1), the“ poor” (category 2), the “intermediate” categories 3 and 4 and the “wealthiest” (category 5). The Wealth Index was constructed from recorded data on household assets such as tables, chairs, refrigerator, air conditioners and beds and also from housing conditions (materials of house floor, house roof, main wall) and facilities (energy for cooking, electricity and latrines), income and expenditure were not used [37].

### Ethical issues

The Scientific Committees of the National Institute of Nutrition (NIN) (Hanoi, Vietnam) and of the Ministry of Health (Hanoi, Vietnam) reviewed and approved the study protocol. All women were informed verbally and in writing about the aims and procedures of the study, and written informed consent was obtained from all women and children, via their mother or guardian approval for these later, before enrollment.

### Statistical analysis

Data entry, including quality checks was performed with Excel 2007™. Data management and analysis were performed with SAS software version 9.2™ (SAS, V9.2; SAS institute, Cary, NC). All analysis took into account characteristics of the cluster sampling design using the appropriate survey procedures of SAS (surveyfreq and surveymeans procedures as introduced in SAS Institute Inc. 2008) [38]. Qualitative variables were expressed as percentages and standard error percentage. Continuous variables were expressed as arithmetic means and standard error of the mean except ferritin whose data were not normally distributed. For, ferritin, concentrations were log-transformed before statistical analysis, and are expressed as geometric means.

Associations between prevalence and region or area (urban/rural) were assessed using univariate logistic regression models (surveylogistic procedure). Associations between continuous variables and region or area were assessed using univariate linear regression models (surveyreg procedure). The first type error rate was set at 0.05.

Logistic regression models with micronutrient prevalence as the response variable were used to assess the effect of the different factors (urban or rural area, socioeconomic class and age class for children) and to estimate Odd Ratios (OR).

## Results

In total, 1526 women and 586 children from 1526 households, 51.6% urban, and 48.4% rural, were surveyed. Among the children, 53.7% were male and 46.3% female. The majority of the surveyed women were from the Kinh ethnic group (86.2%). 15.4% had no education degree, 22.6% had completed primary schooling, 30.7% were secondary school graduates, and 31.3% high school graduates or more. Among households, 16.5% were classified in the “extreme poor” category (category 1), 15.0% in the poor category (category 2), 18.5% in intermediate category 3 and 21.0% in category 4, and 29.1% in the “wealthiest” (category 5).

Anthropometric characteristics of the surveyed women are shown in table 1, respectively, disaggregated by rural and urban strata. The age range of women was 15 to 50 years (Table 1). Among them, 20.5% were classified as underweight, significantly more in rural than in urban area. Less than 10% of the women were classified as overweight or obese, according to the international reference. Using Asian reference, more than 20.0% of the women were classified as overweight or obese. Prevalence of overweight (including obesity) was not significantly different between urban and rural populations with the international reference but significantly higher in the urban population when the Asian reference was applied 22.3% (±1.5%) in urban and 17.9% (±1.4%) in rural area (p<0.05). This double burden is represented in all socioeconomic groups of women with significantly higher overweight and obesity and lower underweight in the two wealthiest groups (p<0.001).

**Table 1:** Nutritional characteristics of women and young children.

	National			Urban					Rural					p
	n****	Mean/Prevalence	SEM/SEP**	n	Mean/Prevalence	SEM/SEP	Adjust. diff/OR***	IC***	n	Mean/Prevalence	SEM/SEP	Adjust. diff/OR	IC	
**Women of reproductive age**														
Age (in years)	1502	32.3	0.3	730	32.7	0.4	0.9	−0.1–1.9	772	31.9	0.3	0.0	-	0.09
Weight (kg)	1502	48.6	0.2	730	49.2	0.3	1.2	0.2–2.1	772	48.0	0.4	0.0	-	0.02
Height (cm)	1501	152.7	0.2	729	152.9	0.3	0.3	−0,5–1.1	772	152.5	0.3	0.0	-	0.42
BMI (kg/m^2^)*	1501	20.8	0.1	729	21.1	0.1	0.4	0.1–0.8	772	20.6	0.1	0.0	-	0.01
] -inf −18.5 [(%)	1501	20.5	1.3	729	17.6	1.5	0.7	0.5–0.9	772	23.3	2.0	1.0	-	0.02
[18.5–25 [(%)	1501	71.5	1.4	729	74.1	1.8	1.3	1.0–1.7	772	69.0	2.1	1.0	-	0.07
[25–30 [(%)	1501	7.4	0.7	729	7.4	1.0	1.0	0.7–1.5	772	7.4	1.1	1.0	-	0.98
[30 - +inf [(%)	1501	0.6	0.2	729	1.0	0.3	3.7	0.8–17.4	772	0.3	0.2	1.0	-	0.09
**young children**														
Age (in months)	565	44.8	0.9	256	45.1	1.0	0.4	−3.0–3.8	309	44.7	1.4	0.0	-	0.81
Weight (kg)	561	13.9	0.2	247	14.4	0.3	1.1	0.3–1.9	307	13.5	0.2	0.0	-	0.02
Height (cm)	563	95.3	0.6	245	96.2	0.8	2.1	−0.3–4.4	303	94.5	0.9	0.0	-	0.14
WHZ*	557	−0.4	0.1	250	−0.2	0.1	0.4	0.1–0.6	307	−0.6	0.1	0.0	-	0.01
wasting (%)	557	6.3	1.2	250	5.2	1.8	0.7	0.3–1.7	307	7.2	1.7	1.0	-	0.44
WAZ*	558	−1.0	0.1	250	−0.7	0.1	0.5	0.1–0.8	308	−1.2	0.1	0.0	-	<0.01
underweight (%)	558	18.1	2.0	250	14.8	2.2	0.7	0.4–1.1	308	20.8	3.2	1.0	-	0.11
HAZ*	552	−1.1	0.1	249	−0.9	0.1	0.4	0.1–0.7	303	−1.3	0.1	0.0	-	0.02
stunting (%)	552	23.2	2.6	249	18.9	3.5	0.6	0.4–1.1	303	26.7	3.6	1.0	-	0.13

* :Body Mass Index (BMI), weight-for-age<-2z-scores (WAZ) for underweight, height-for-age<-2 z-scores (HAZ) for stunting and weight-for-height<-2 z-scores (WHZ) for wasting.

** SEP: standard of error of the prevalence; SEM: standard error of the mean.

*** : IC: interval of confidence; OR: Odd Ratio.

**** few children (21 over 586) and women (24 over 1526) did not have any anthropometric measurements.

Micronutrient status of women is also presented in table 2. Anemia prevalence was affecting approximately 12% of women; Prevalence of ID was present in approximately 14% of women, with 5.4% of the women classified as IDA. The prevalence of zinc deficiency was much higher, affecting two-third of the women. The prevalence of vitamin A deficiency was <2% in women whereas approximately 14% had a marginal vitamin A status. Prevalence of folate deficiency was <3% whereas marginal folate status affected one fourth of women. Prevalence of vitamin B_12_ deficiency was 12%, with an additional 4% of the women having a marginal status.

**Table 2:** Vitamins and mineral status indicators and prevalence of deficiencies among women of reproductive age.

	Global			Urban					Rural					p
	n***	Mean/Prevalence	SEM/SEP	n	Mean/Prevalence	SEM/SEP	Adjust. diff/OR	IC	n	Mean/Prevalence	SEM/SEP	Adjust. diff/OR	IC	
Hb (g/l)	1526	131.4	0.4	740	131.7	0.5	0.1	−0.1–0.2	786	131.1	0.5	0.0	-	0.39
Iron status														
Anemia (%)	1526	11.6	1.0	740	11.0	1.4	0.9	0.6–1.3	786	12.2	1.6	1.0	-	0.54
Ferrintin (µg/l)*	1523	48.5	1.0	739	60.0	1.1			784	45.5	1.1			0.06
Iron deficiency (%)	1523	13.7	1.1	739	11.8	1.3	0.7	0.5–1.0	784	15.6	1.8	1.0	-	0.09
Iron deficiency anemia (%)	1522	5.4	0.7	738	4.6	0.8	0.7	0.5–1.2	784	6.1	1.0	1.0	-	0.24
Vitamin A status														
Plasma retinol (µmol/l)	1475	1.49	0.02	706	1.48	0.03	−0.02	−0.11–0.06	769	1.50	0.03	0.00	-	0.58
Vitamin A deficiency (%)	1475	1.6	0.3	706	1.6	0.5	1.0	0.4–2.3	769	1.6	0.4	1.0	-	0.99
Marginal status (%)	1475	13.6	1.0	706	14.2	1.5	1.1	0.8–1.5	769	13.1	1.4	1.0	-	0.62
Zinc status														
Plasma zinc (µmol/l)	1522	9.5	0.1	739	9.7	0.2	0.4	−0.1–0.9	783	9.3	0.2	0.0	-	0.13
Zinc deficiency (%)	1522	67.2	2.6	739	62.9	3.8	0.7	0.4–1.1	783	71.1	3.6	1.0	-	0.12
Folate status														
Plasma folate (nmol/l)**	1472	17.6	0.6	705	16.5	0.8	−1.8	−5.7–2.1	767	18.7	0.7	0.0	-	
Folate deficiency (%)	1472	2.7	0.6	705	2.8	0.6	1.1	0.5–2.7	767	2.5	0.9	1.0	-	0.75
Marginal status (%)	1472	25.1	1.6	705	27.7	2.5	1.3	0.9–1.8	767	22.8	2.2	1.0	-	0.14
Vitamin B12 status														
Plasma B12 (pmol/l)	505	630.3	23.1	230	668.9	33.4	70.9	−20.1–164.8	275	598.0	31.4	0.0	-	0.13
B12 deficiency (%)	505	11.7	1.7	230	11.7	2.5	1.0	0.5–1.9	275	11.6	2.2	1.0	-	0.97
Marginal status (%)	505	3.8	1.1	230	2.2	1.0	0.4	0.1–1.3	275	5.1	1.8	1.0	-	0.13
Inflammation														
CRP (mg/l)	1518	1.8	0.1	736	2.1	0.2	0.5	0.0–1.0	782	1.6	0.1	0.0	-	0.03
High CRP (%)	1518	6.8	0.6	736	8.0	0.9	1.5	1.0–2.2	782	5.6	0.9	1.0	-	0.07

* geometric mean.

** median values.

*** The sample size varied slightly for each micronutrient analysis because of insufficient blood quantity among some participants.

**Figure 1:**
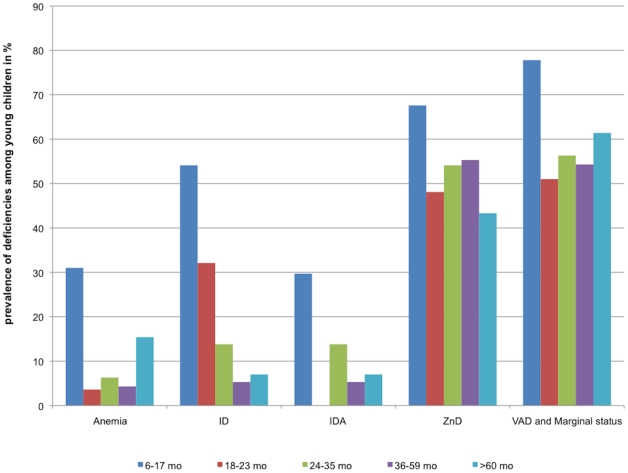
Prevalence of micronutrient deficiencies among young children by age groups (in %). ID: iron deficiency; IDA: iron deficiency anemia, ZnD: zinc deficiency, VAD: vitamin A deficiency; mo: months.

The age range of children was 10 to 75 months: 23% was stunted and 6% wasted. In contrast, 7% of the children were overweight or obese. Anemia prevalence was close to 10%, ID was 13% with 3% of the children having IDA (Table 3). The prevalence of zinc deficiency was high, affecting more than half of the children, and significantly more prevalent in rural than in urban areas. The prevalence of Vitamin A deficiency was 10% whereas almost half of children had a marginal vitamin A status. Folate deficiency was less than 1% in children and marginal folate approximately 6%, with urban children being more affected by marginal-deficient status than rural children (p<0.05). Figure 1 shows the prevalence of the micronutrient deficiencies according to the age. There was a clear effect of age on anemia prevalence and iron status, with the youngest age group (6–17 mo) having the highest risk for anemia and IDA as compared to the other groups (table S1). For zinc and vitamin A deficiency, age was a less strong factor, although the risk for deficiency or marginal status was highest in the youngest age group.

**Table 3:** Vitamins and mineral status indicators and prevalence of deficiencies among young children.

	Global			Urban					Rural					p
	n***	Mean/Prevalence	SEM/SEP	n	Mean/Prevalence	SEM/SEP	Adjust. diff/OR	IC	n	Mean/Prevalence	SEM/SEP	Adjust. diff/OR	IC	
Hb (g/l)	583	125.3	0.1	262	125.8	0.1	0.1	−0.2–0.3	321	125.0	0.1	0.0	-	0.51
Iron status														
Anemia (%)	583	9.1	1.4	262	8.0	2.1	0.8	0.4–1.6	321	10.0	1.9	1.0	-	0.50
Ferrintin (µg/l)*	568	33.8	1.0	259	32.1	1.1			309	35.3	1.1			0.21
Iron deficiency (%)	568	12.9	1.5		14.7	2.4	1.3	0.8–2.3		11.3	2.0	1.0	-	0.27
Iron deficiency anemia (%)	564	3.2	0.8	258	1.9	1.2	0.4	0.1–1.8	306	4.3	1.1	1.0	-	0.25
Vitamin A status														
Plasma retinol (µmol/l)	546	1.04	0.02	242	1.06	0.04	0.05	−0.05–0.15	304	1.02	0.03	0.00	-	0.35
Vitamin A deficiency (%)	546	10.1	1.6	242	8.7	2.3	0.8	0.4–1.5	304	11.2	2.2	1.0	-	0.43
Marginal status (%)	546	47.3	2.2	242	42.6	3.2	0.7	0.5–1.0	304	51.0	3.1	1.0	-	0.06
Vitamin A deficiency and Marginal(%)	546	57.3	2.7	242	51.2	3.4	0.6	0.4–1.0	304	62.2	3.9	1.0	-	0.04
Zinc status														
Plasma zinc (µmol/l)	563	10.2	0.2	257	10.8	0.3	1.1	0.3–1.8	306	9.7	0.2	0.0	-	<0.01
Zinc deficiency (%)	563	51.9	3.5	257	42.0	5.0	0.5	0.3–0.8	306	60.1	4.7	1.0	-	<0.01
Folate status														
Plasma folate (nmol/l)**	327	25.4	1.1	147	22.3	1.7	−4.5	−17.0–8.0	180	26.7	1.3	0.0	-	0.48
Folate deficiency (%)	327	0.6	0.4	147	1.4	0.9	-	-	180	0	-	1.0	-	<0.01
Marginal status (%)	327	6.4	1.5	147	9.5	2.7	2.6	0.9–7.1	180	3.9	1.5	1.0	-	0.06
Folate deficiency and Marginal(%)	327	7.0	1.6	147	10.9	3.1	3.0	1.1–8.3	180	3.9	1.5	1.0	-	0.03
Inflammation														
CRP (mg/l)	566	2.7	0.5	258	2.0	0.3	−1.3	−3.0–0.5	308	3.3	0.8	0.0	-	0.16
High CRP (%)	566	10.8	1.4	258	11.2	2.0	1.1	0.6–1.9	308	10.4	2.0	1.0	-	0.76

* geometric mean.

** median values.

*** The sample size varied slightly for each micronutrient analysis because of insufficient blood quantity among some participants.

**Figure 2:**
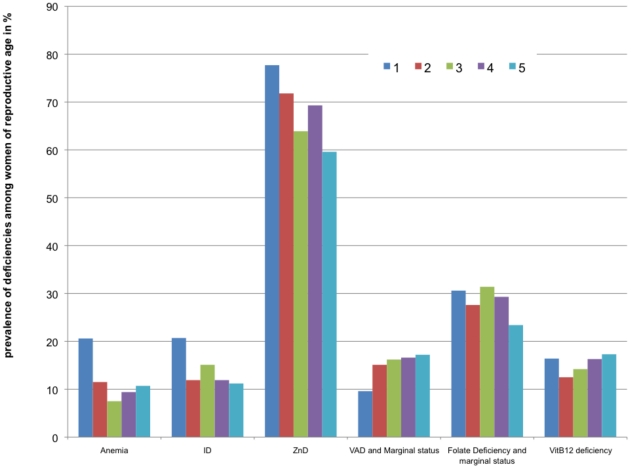
Prevalence of micronutrient deficiencies among women of reproductive age by socioeconomic groups* (in %). *note: Socio-economic categories: 1: the “extreme poor”; 2: the “poor”, 3 and 4: the “intermediate” and 5: the “wealthiest”. Sample size respectively for category 1,2,3,4 and 5: Anemia (n = 248, 226, 279, 319 and 440); ID (n = 246, 227, 278, 319, 439); Vitamin A deficiency and marginal status (n = 240, 219, 260, 313, 429); ZD (n = 247, 227, 277, 319, 438); folate deficiency and marginal status (n = 245, 221, 261, 304, 428); vitamin B12 deficiency and marginal status (n = 67, 80, 106, 98, 150).

**Figure 3:**
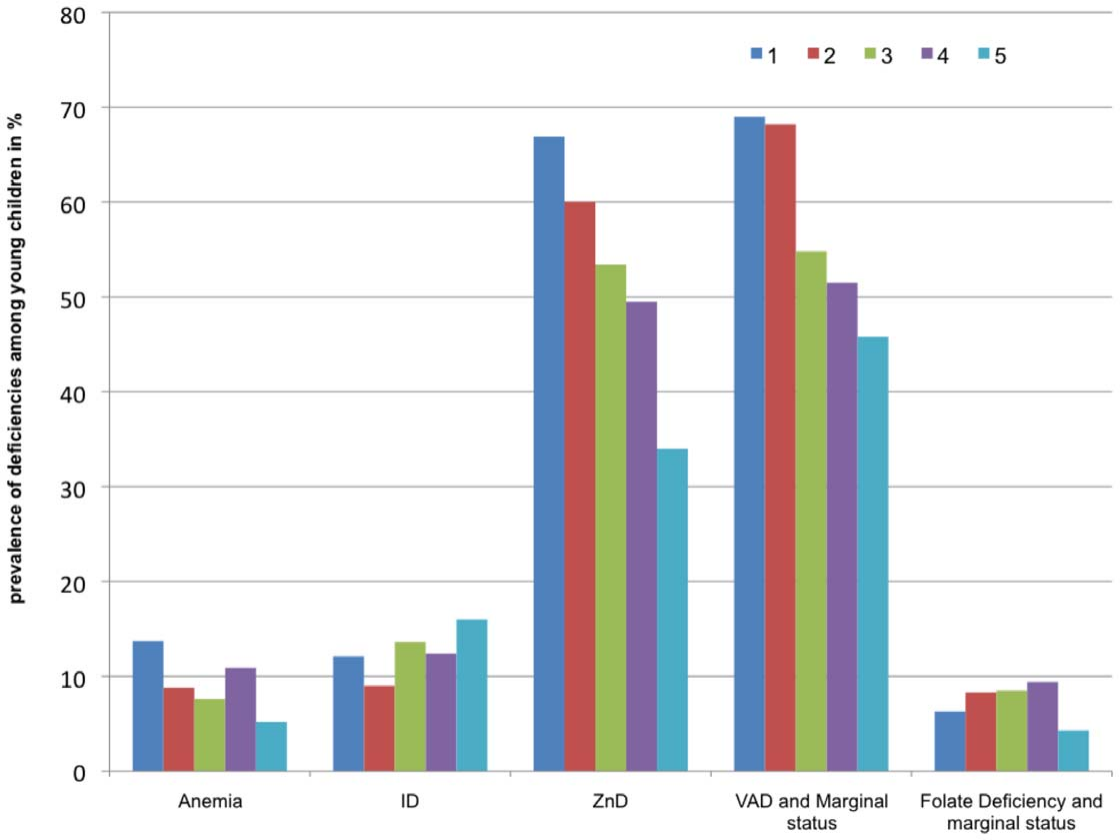
Prevalence of micronutrient deficiencies among young children by socioeconomic groups* (in %). *note: Socio-economic categories: 1: the “extreme poor”; 2: the “poor”, 3 and 4: the “intermediate” and 5: the “wealthiest”. Sample size respectively for category 1,2,3,4 and 5: Anemia (n = 131, 91, 92, 110, 154); ID (n = 132, 89, 88, 105, 150); Vitamin A deficiency and marginal status (n = 129, 88, 84, 97, 144); ZD (n = 130, 89, 88, 103, 150); folate deficiency and marginal status (n = 63,48, 59, 64, 93).

Data were also analyzed by socio-economic (SE) categories. Women in the poorest category had approximately 2 times higher risk for anemia and ID than women in the other categories (table 4 and figure 2). Zinc deficiency showed a trend going in the same direction with prevalence decreasing from 77% to 60% from the poorest to the wealthiest category whereas the risk for vitamin A marginal status was in contrast significantly lower in the poorest category 1. The risks for folate or vitamin B12 deficiencies were not significantly different among SE categories. In children (table 5 and figure 3), the risk for zinc deficiency and vitamin A deficiency and marginal status was significantly higher in the poorest category.

**Table 4:** Prevalence of vitamins and mineral deficiencies among women by socioeconomic categories.

	1			2					3					4					5					p
	n	Mean/Prev.	SEM/SEP*	n	Mean/Prev.	SEM/SEP	Adjust. diff/OR*	IC	n	Mean/Prev.	SEM/SEP	Adjust. diff/OR	IC	n	Mean/Prev.	SEM/SEP	Adjust. diff/OR	IC	n	Mean/Prev.	SEM/SEP	Adjust. diff/OR	IC	
Iron status																								
Anemia (%)	248	20.6	3.0	226	11.5	2.3	0.5	0.3–0.9	279	7.5	1.7	0.3	0.2–0.6	319	9.4	1.7	0.4	0.2–0.7	440	10.7	1.7	0.5	0.3–0.8	<0.01
Iron deficiency (%)	246	20.7	3.4	227	11.9	2.7	0.5	0.3–0.9	278	15.1	2.1	0.7	0.4–1.1	319	11.9	2.0	0.5	0.3–0.9	439	11.2	1.6	0.5	0.3–0.8	0.04
Vitamin A status																								
Vitamin A deficiency (%)	240	2.1	0.9	219	0	-	-	-	260	1.5	0.7	0.7	0.2–2.2	313	1.6	0.7	0.8	0.2–2.6	429	2.1	0.7	1.0	0.3–3.1	<0.01
Marginal status (%)	240	7.5	1.6	219	15.1	2.7	2.2	1.2–4.0	260	14.6	2.3	2.1	1.2–3.6	313	15.0	2.0	2.2	1.3–3.8	429	15.2	2.0	2.2	1.3–3.7	0.03
Zinc status																								
Zinc deficiency (%)	247	77.7	3.8	227	71.8	4.1	0.7	0.4–1.2	277	63.9	4.5	0.5	0.3–0.8	319	69.3	3.5	0.6	0.4–1.1	438	59.6	4.0	0.4	0.2–0.7	<0.01
Folate status																								
Folate deficiency (%)	245	3.7	1.3	221	2.7	1.2	0.7	0.2–2.2	261	3.8	1.5	1.0	0.4–2.9	304	1.6	0.7	0.4	0.1–1.4	428	1.9	0.7	0.5	0.2–1.4	0.37
Marginal status (%)	245	26.9	2.8	221	24.9	3.2	0.9	0.6–1.3	261	27.6	3.1	1.0	0.7–1.5	304	27.6	3.5	1.0	0.7–1.6	428	21.5	2.4	0.7	0.5–1.1	0.27
Vitamin B12 status																								
B12 deficiency (%)	67	10.4	3.9	80	10.0	3.5	1.0	0.3–3.1	106	10.4	3.6	1.0	0.3–3.0	98	14.3	3.4	1.4	0.5–3.9	150	12.7	2.7	1.2	0.5–3.2	0.79
Marginal status (%)	67	6.0	2.5	80	2.5	2.4	0.4	0.1–2.2	106	3.8	2.3	0.6	0.1–2.9	98	2.0	1.4	0.3	0.1–1.7	150	4.7	1.7	0.3	0.1–1.7	0.55

* SEP: standard of error of the prevalence; SEM: standard error of the mean; IC: interval of confidence; OR: Odd Ratio.

**Table 5:** Prevalence of vitamins and mineral deficiencies among children by socioeconomic categories.

	1			2					3					4					5					p
	n	Mean/Prev.	SEM/SEP*	n	Mean/Prev.	SEM/SEP	Adjust. diff/OR*	IC	n	Mean/Prev.	SEM/SEP	Adjust. diff/OR	IC	n	Mean/Prev.	SEM/SEP	Adjust. diff/OR	IC	n	Mean/Prev.	SEM/SEP	Adjust. diff/OR	IC	
Iron status																								
Anemia (%)	131	13.7	3.2	91	8.8	3.2	0.6	0.2–1.5	92	7.6	2.7	0.5	0.2–1.2	110	10.9	3.4	0.8	0.3–1.8	154	5.2	2.3	0.3	0.1–1.0	0.21
Iron deficiency (%)	132	12.1	3.4	89	9.0	3.4	0.7	0.2–2.1	88	13.6	3.5	1.1	0.5–2.6	105	12.4	3.3	1.0	0.4–2.5	150	16.0	3.4	1.4	0.6–3.0	0.68
Vitamin A status																								
Vitamin A deficiency (%)	129	14.0	3.6	88	6.8	3.1	0.5	0.2–1.4	84	6.0	2.6	0.4	0.1–1.1	97	13.4	3.9	1.0	0.4–2.2	144	9.0	2.5	0.6	0.3–1.4	0.29
Marginal status (%)	129	55.0	4.7	88	61.4	7.0	1.3	0.6–2.7	84	48.8	5.6	0.8	0.5–1.3	97	38.1	5.4	0.5	0.3–0.9	144	36.8	3.7	0.5	0.3–0.8	<0.01
Zinc status																								
Zinc deficiency (%)	130	66.9	5.1	89	60.0	6.9	0.7	0.4–1.4	88	53.4	6.6	0.6	0.3–1.1	103	49.5	6.1	0.5	0.3–0.9	150	34.0	5.3	0.3	0.1–0.5	<0.01
Folate status																								
Folate deficiency (%)	63	0	-	48	0	-	1	0.6–1.7	59	1.7	1.7	-	-	64	0		1	0.6–1.7	93	1.1	1.1	-	-	<0.01
Marginal status (%)	63	6.3	3.5	48	8.3	3.6	1.3	0.3–6.7	59	6.8	4.0	1.1	0.2–6.0	64	9.4	4.3	1.5	0.3–6.9	93	3.2	1.8	0.5	0.1–2.5	0.67

* SEP: standard of error of the prevalence; SEM: standard error of the mean; IC: interval of confidence; OR: Odd Ratio.

## Discussion

This study showed that according to the WHO classification [30] the prevalence of anemia was a mild public health problem ([5.0–20[% [39]) in both the women of reproductive age and children aged from 0.5 to 7 years. Vitamin A deficiency was also recognized as a mild public health problem (≥10% of the population) among young children as more than 10% were deficient [30], [40] but not in women. Zinc deficiency was notably high in both women and children representing a severe public health concern (≥25% among children under 5 years of age) [41]. Folate deficiency was not of concern in both population groups whereas the prevalence of low serum vitamin B12 among women, two times higher than the cut-off of 5%, pointed out a country-wide public health problem [42].

In the 2000 “National Anemia, Nutrition and Risk Factor Survey”, anemia prevalence in Vietnam was 34% in children under five years of age and 25% in women of reproductive age [43]. In the present study, the prevalence of anemia in women and children is much less, suggesting a substantial improvement in the prevalence of anemia over the last decade. Several factors might have contributed to these findings. Firstly, the health authorities in Vietnam have included the prevention of iron deficiency in their 2001–2010 National Plan of Action for Nutrition [44]. The strategy focused on reducing child malnutrition and low birth weights, and also vitamin A, iron and iodine deficiencies through supplementation, and food diversification and fortification. Secondly, from the late 1990's to 2005, the *per capita* income in Vietnam increased from US$300 to more than US$400 [45], [46], [47], and child malnutrition was improved with a decline from 1990 to 2010 of stunting from 56.5% to 29.3% and underweight from 51.5% to 17.5% [6], [48]. Evidence from South East Asia shows that economic growth generally leads to improvement in human nutrition [45]. In Vietnam, the increase of income since the 1990's is associated with an increased consumption of meats, fish and fruits [49] which are mainly rich source of heme iron and/or enhancing factors for non-heme iron absorption [50].

We cannot discard the possibility that the method used in this study to measure hemoglobin concentrations has impacted the results. Indeed, in the present study, the hemoglobin concentration was measured with the new HemoCue Hb301® device and a recent study showed that this device yields slightly higher values (+0.5 g/dL) than the Hemocue Hb201® device used in most of the previous studies in Vietnam [51]. However, using cut-offs for anemia increased by 5 g/L to correct for this, anemia would be present in 16.0% (CI: 12.3–19.8 g/L%) of the children and 21.4% (CI: 19.0–23.8%) of the women without significant difference between urban and rural populations. Hence, still lower than the anemia prevalence data from 2000.

The measure of iron stores, that is not often included in national surveys, indicated that the prevalence of iron deficiency was also low and associated to approximately half or less of the anemia cases, suggesting others causes than iron for anemia, such as other micronutrients deficiencies (vitamin A, folate or VitB_12_) or hemoglobinopathies as malaria was not an important issue in the present study. Hemoglobinopathies can cause a shift to the left in the Hb distribution, leading to more people having hemoglobin concentrations below the relevant cut-offs, without having clear physiological consequences. In addition, chronic inflammation (e.g. intestinal parasites) can lead to anemia through reduced erythropoiesis (a reduction in red blood cell production by the bone marrow). Unfortunately, we do not have data to identify which of these factors underlies the anemia in our cohort. Moreover, half of the ID was not associated with anemia suggesting that daily iron intake in these subjects was sufficient to allow normal physiological functions but not adequate to build iron stores. Over 25% of children and women had a ferritin concentration <30 µg/l that indicates low iron stores (less than 180–200 mg of iron). This is especially important for women of reproductive age as iron stores of 300–500 mg before pregnancy are recommended [52]. Anemia has many etiologies, iron deficiency being only one of them.

In children, the stratification of anemia, ID and IDA by age group showed that the prevalence and risk of anemia, iron deficiency and iron deficiency anemia was significantly higher in the 6–17 mo. age group. In this age group, the prevalence of anemia was considered as a moderate public health problem and most of anemia was associated with iron deficiency. More than half of children had ID, a finding consistent with intervention trials carried out in previous years in Vietnamese infants and young children [14], [53], [54]. In older children risk for anemia and ID was much lower, although still high for the 18–23 mo group (∼30%). A recent situational review of infant and young child feeding practices in Viet Nam showed that exclusive breastfeeding until 6 months is rare in Vietnam [55]. The article also suggested that complementary foods for young children have low energy density, low protein and micronutrient content as rice flour and rice porridge are the most common used food, which might increase the risk of iron deficiency anemia. The strong effect of age on anemia and iron status is probably due to access of the children to a more diversified diet, perhaps with fortified products such as fish sauce, and to lower iron needs [56].

Very few countries have national data on zinc deficiency measured through plasma zinc. Indeed, most of data on zinc deficiency are obtained from prevalence of zinc intakes below the EAR, and of stunted children less than 5 years of age [41], [57], [58]. Although plasma zinc is often questioned as a marker for individual zinc status, it is still a reliable marker on population level [59], [60]. One of the main concerns with plasma zinc is the potential contamination of blood samples that may lead to underestimate the prevalence of zinc deficiency. In this survey material and procedure were controlled for contamination. The high prevalence of zinc deficiency in both women and children in this survey is intriguing, although consistent with several intervention trials carried-out in Vietnam in the recent years which indicated zinc deficiency prevalence ranging from 32% to 67% in young children under 2 years of age [14], [54] and of 52.1% among rural primary schoolchildren aged 6 to 8 years of age [8]. Moreover, according to a recent review assessing the risk of zinc deficiency, South and Southeast Asia, appear to be at the highest risk of zinc deficiency [59]. In Southeast Asia, 6 over 10 evaluated countries were categorized as high risk countries for zinc deficiency, including Vietnam [61]. Considering children under 2 years of age, 30.1% were stunted and 56.2% had zinc deficiency. This would suggest that data on stunting, without zinc dietary assessment, as a proxy for zinc deficiency is not relevant. In addition, stunting is a cumulative phenomenon due to many causes and it is difficult to link stunting to a single measure of plasma zinc.

Vitamin A deficiency as well as marginal vitamin A status was low in women and much higher in children. The most recent survey conducted in 2000 in 4 ecological regions of Vietnam showed a prevalence of VAD in children under 2 years of age of 15% [62]. Now, a decade later, VAD prevalence was not notably lower as 11.8% in the children under two years of age, and 11.9% of the children between 2 to 5 years of age were VAD deficient. More than half of children had a marginal or deficient vitamin A status with a significant higher risk for the youngest. This high prevalence of marginal vitamin A status would suggest that efforts to prevent vitamin A deficiency have to be maintained and even strengthened especially for the under 2 year old children. Eliminating vitamin A deficiency through vitamin A supplementation has been the focus of universal efforts. However, two studies in the Philippines and India show that the effect of high dose vitamin A capsules last between 2–3 mo with no more effect after 6 mo. [63], [64]. This would suggest that other complementary types of intervention, such as food fortification or food diversification should be planed and evaluated.

Very few countries, especially from Asia have national data on folate or vitamin B_12_ status [42]. The low prevalence of folate deficiency in women in this survey is in agreement with a recent study conducted in 2006 among women living in Hanoi and Hai Phuong provinces that did not show evidence of folate and vitamin B_12_ deficiencies [11]. However, approximately one third of women in our survey had a marginal folate status. In the 2006 study, suboptimal folate status, measured on red blood cell folate concentration, was also much higher (60%) than folate deficiency, warranting special attention to ways to improve folate status also. Our study may also suggest that marginal folate status is affecting women more than young children, a finding not described before. In contrast with the 2006 study, vitamin B_12_ deficiency represented a public health problem in our survey. This situation could place the future babies at increased risk for neural tube defects as deficient or inadequate maternal vitamin B_12_ and folate are associated with a significantly increased risk for neural tube defects [65], [66]. The marginal folate and vitamin B_12_ status found here and the fact that adequate folate and vitamin B_12_ status one month before pregnancy and during the first trimester of pregnancy are strongly recommended [67] suggest that preventive measures to increase intakes of folate and B_12_ should be taken into consideration.

Prevalence of micronutrient deficiencies differed according to socio-economic levels. The highest risk for anemia, iron and zinc deficiency in the poorest category of women and children was probably due to the lowest consumption of food containing bio-available iron and zinc such as meat or fish as demonstrated in a recent study [68]. Risk of marginal vitamin A status was also significantly higher among the poorest children but surprisingly lower among women. At this stage, we cannot propose any explanation until the analysis of food consumption data has been completed.

This survey showed also that Vietnam is facing the so-called double burden of malnutrition, with on one side, women with overweight (20%) and on the other side women with underweight (20%) or/and micronutrient deficiencies. Those results confirms previous findings which emphasize the Vietnamese nutrition transition [7]. This double burden is represented in all socioeconomic groups of women with significantly higher overweight and obesity and lower underweight in the two higher wealthiest groups. This survey shows that overweight and obesity, measured with international reference, increased over the last decade. The two last Vietnamese Living Standard Surveys *(VLSS)* show that the prevalence of overweight in Vietnamese adults doubled between 1992 and 2002, from 2% to 5.5%, respectively [69], and to 8% in this survey. Moreover, the prevalence of overweight and obesity measured with the Asian BMI cut-off in our study was higher (22.4%) than in the 2005 National Adult Obesity survey that was 16.3% [70]. In contrast, the prevalence of underweight did not change notably between 2005 and now. In this survey, the prevalence of overweight (including obesity) was significantly higher in the urban population. This is reinforced by the prevalence of 33.2% of overweight and obesity found in 2005 in a representative sample of 1,971 adults aged 25–64 years living in Ho Chi Minh City (HCMC) [71]. The magnitude of overweight (measured with international cut-offs) is still much less in Vietnam than in many of the neighboring countries. In Thailand, the National Health Examination Survey II showed a prevalence of overweight in adults aged 20–59 years of 28%, with highest values in women (33.9%) and in the urban population (34.8%) [72]. The problem of overweight and obesity is also rapidly increasing in China in all gender and age groups and in geographical areas, particularly in the urban area, with overall reported prevalence rates of 15% in 1992 and 22% in 2002 [73]. Other countries in the region are also hit by over-nutrition as 29% of the adults in Hong Kong [74] and 26.7% in South-Korea [75] are classified overweight or obese.

### Conclusion

One of the main new findings of this survey was that zinc deficiency is a severe public health problem among women in reproductive age and in children age 6–75 mo. In women, Vitamin B_12_ deficiency was also a concern. In contrast, in both population groups, the prevalence of anemia, iron and vitamin A deficiency have decreased compared to previous surveys. However, marginal anemia and marginal vitamin A status are still prevalent. The other main finding was that the group of children from 6 to 18 months of age was particularly at risk for micronutrient deficiencies. Consequently specific interventions for this age group to improve food diversity and quality should be implemented. Among them, the micronutrient fortification of staple foods and condiments as well as special attention to complementary feeding could contribute to prevent the Vietnamese population from becoming micronutrient deficient, and need to be explored.

## Supporting Information

Table S1 Prevalence of vitamins and mineral deficiencies for children by age groups*. *SEP: standard of error of the prevalence; SEM: standard error of the mean; IC: interval of confidence; OR: Odd Ratio.
(DOCX)
