# Supplementary material for: Micronutrient Deficits Are Still Public Health Issues among Women and Young Children in Vietnam
Source: PLoS One. 2012 Apr 17;7(4):e34906. doi: 10.1371/journal.pone.0034906 (PMC3328495; doi:10.1371/journal.pone.0034906)
Supplement: Table S1 — Prevalence of vitamins and mineral deficiencies for children by age groups*. *SEP: standard of error of the prevalence; SEM: standard error of the mean; IC: interval of confidence; OR: Odd Ratio. (DOCX) [file pone.0034906.s001.docx]

Table S1. Prevalence of vitamins and mineral deficiencies for children by age groups*.

|  | ]-inf - 18] | | | ]18 - 24] | | | | | ]24 - 36] | | | | | ]36 - 60] | | | | | ]60 - +inf] | | | | | p |
| --- | --- | --- | --- | --- | --- | --- | --- | --- | --- | --- | --- | --- | --- | --- | --- | --- | --- | --- | --- | --- | --- | --- | --- | --- |
|  | n | Mean / Prev. | SEM / SEP | n | Mean / Prev. | SEM / SEP | Adjust. diff/ OR | IC | n | Mean / Prev. | SEM / SEP | Adjust. diff/ OR | IC | n | Mean / Prev. | SEM / SEP | Adjust. diff/ OR | IC | n | Mean / Prev. | SEM / SEP | Adjust. diff/ OR | IC |  |
| Iron status |  |  |  |  |  |  |  |  |  |  |  |  |  |  |  |  |  |  |  |  |  |  |  |  |
| Anemia (%) | 42 | 31.0 | 7.7 | 56 | 3.6 | 2.5 | 0.1 | 0.0-0.4 | 111 | 6.3 | 2.6 | 0.2 | 0.0-0.5 | 209 | 4.3 | 1.7 | 0.1 | 0.0-0.2 | 143 | 15.4 | 3.3 | 0.4 | 0.2-0.9 | <0.01 |
| Iron deficiency (%) | 37 | 54.1 | 9.7 | 53 | 32.1 | 6.5 | 0.4 | 0.1-1.1 | 109 | 13.8 | 3.2 | 0.1 | 0.1-0.3 | 207 | 5.3 | 1.8 | 0.0 | 0.0-0.1 | 143 | 7.0 | 2.0 | 0.1 | 0.0-0.2 | <0.01 |
| Iron deficiency anemia (%) | 37 | 29.7 | 8.3 | 53 | 0 | 0 | - | - | 106 | 0.9 | 1.0 | 0.0 | 0.0-0.2 | 207 | 1.0 | 0.7 | 0.0 | 0.0-0.1 | 142 | 2.8 | 1.3 | 0.1 | 0.0-0.2 | <0.01 |
| Zinc deficiency (%) | 37 | 67.6 | 9.3 | 52 | 48.1 | 8.0 | 0.4 | 0.2-1.3 | 109 | 54.1 | 5.5 | 0.6 | 0.2-1.3 | 206 | 55.3 | 4.5 | 0.6 | 0.2-1.4 | 141 | 43.3 | 5.3 | 0.4 | 0.1-0.9 | 0.11 |
| Vitamin A status |  |  |  |  |  |  |  |  |  |  |  |  |  |  |  |  |  |  |  |  |  |  |  |  |
| Vitamin A deficiency (%) | 36 | 13.9 | 5.8 | 49 | 10.2 | 5.0 | 0.7 | 0.2-2.9 | 103 | 15.5 | 4.8 | 1.1 | 0.3-3.8 | 199 | 10.1 | 2.2 | 0.7 | 0.2-2.0 | 140 | 5.0 | 1.8 | 0.3 | 0.1-1.1 | 0.06 |
| Marginal status (%) | 36 | 63.9 | 8.4 | 49 | 40.8 | 7.1 | 0.4 | 0.2-0.9 | 103 | 40.8 | 4.9 | 0.4 | 0.2-0.9 | 0.9 | 44.2 | 3.5 | 0.4 | 0.2-1.0 | 140 | 56.4 | 4.1 | 0.7 | 0.3-1.6 | 0.03 |

*SEP: standard of error of the prevalence; SEM: standard error of the mean; IC: interval of confidence; OR: Odd Ratio
